# Calcineurin/CXCR4 in T-ALL

**DOI:** 10.18632/oncoscience.238

**Published:** 2015-09-12

**Authors:** Diana Passaro, Christine Tran Quang, Jacques Ghysdael

**Affiliations:** Institut Curie, Centre Universitaire, Orsay, France

**Keywords:** acute lymphoblastic leukemia, CXCR4, CXCL12, calcineurin, targeted therapy

The calcineurin/NFAT signaling pathway is implicated in a wide variety of biological processes, acting as a bridge pathway between calcium signals and gene expression. Although its role as an effector of immune responses figured prominently in early studies, it forms just one part of a larger picture. Indeed calcineurin has been shown to participate in the development and function of e.g. the immune, cardiovascular, nervous and musculoskeletal systems, and dysregulation of calcineurin/NFAT signaling contributes to pathologies affecting these tissues, in particular cancer [1].

Activation of the calcineurin/NFAT pathway was first observed in human lymphoma, as well as in mouse models of T cell acute lymphoblastic leukemia (T-ALL) and xenotransplanted human T-ALL [2]. In T-ALL, calcineurin activation is independent of preTCR/TCR signaling (the major calcineurin activator in normal T cell progenitors), but strongly depends upon micro-environmental signals. The fundamental, intrinsic role of calcineurin in T-ALL was demonstrated in several mouse models, in which conditional calcineurin genetic deletion was restricted to leukemic cells. Calcineurin was found essential to the physical and functional interactions that leukemic cells establish with supportive stromal cells, with its deletion resulting in impaired leukemia propagation, reduced cell survival, proliferation, migration and homing [3]. The therapeutic relevance of these findings was highlighted by preclinical studies showing strong anti-leukemic effects of calcineurin inhibitors (namely cyclosporin A or tacrolimus [FK506]) and long-term leukemia remission in a mouse T-ALL model when vincristine treatment was combined with calcineurin genetic inactivation [2, 3]. However, available calcineurin inhibitors appear suboptimal as potential therapeutic agents since they are associated with a number of toxic side effects [4], show clear off-target effects in T-ALL cells [3] and are expected to interfere with the anti-tumor immune response.

A recently developed, alternative option is to identify and target molecular pathways acting downstream of calcineurin and critical to T-ALL maintenance [5]. Our global transcriptomic analysis identified a large number of calcineurin-dependent genes in T-ALL, involved in an array of biological function, including the de-repression of known tumor suppressive pathways (e.g. CDKN1A) [3]. Although of high biological interest, these deregulations are not easily accessible for targeted therapy. In contrast, genes/proteins implicated in the adhesion/migration to the bone marrow microenvironment are promising candidates (i) for a thorough understanding of the factors that contribute to microenvironment-mediated support of leukemia progression and (ii) for the design of niche-targeted therapies. Along these lines, we linked calcineurin-dependent regulation of the adhesive/migratory properties of T-ALL cells to a boost of CXCR4 surface expression and the subsequent ability of the leukemic cells to respond to CXCL12 [5]. Upregulation of CXCR4 cell surface expression was also demonstrated in diagnostic T-ALL cases and primary xenograft in NSG mice [5][6]. The mechanism by which calcineurin affects CXCR4 trafficking is partially explained by the Cn-dependent up-regulation of cortactin [5], an actin-binding protein implicated in the regulation of endosomal trafficking [7]. Because actin polymerization is required for CXCR4 and other chemokine receptors trafficking to recycling vesicles, inhibition of cortactin expression in calcineurin-deficient T-ALL cells likely results in impaired actin dynamics in this endosomal compartment. Further investigation of the intrinsic function of CXCR4 in murine and human T-ALL revealed an important role of CXCL12/CXCR4 signaling in both survival/proliferation and homing/migration of leukemic cells to the supportive bone marrow niche [5][6]. Intravital multiphoton imaging and genetic studies revealed a strong interaction between T-ALL cells and CXCL12-expressing niche(s), and an essential supportive function of CXCL12 produced by vascular endothelial cells [6]. Local CXCL12 production, in addition to induction of CXCR4-dependent signaling cues will result in activation of T-ALL cells specific integrins, further stabilizing adhesion to integrin ligands expressing niche(s) and induction of additional pro-survival signals. In this scenario, the nature of the niche cells expressing the integrin ligands requires further characterization.

Strikingly, CXCR4 is also critical to leukemia initiating cell activity (LIC) in murine T-ALL and human xenografts [5][6], highlighting an unexpected, fundamental function of microenvironmental signals for T-ALL maintenance and progression. Many inhibitors of CXCL12 or CXCR4 have been developed and are tested in clinical studies in other pathological contexts, in particular other hematological malignancies [8]. However, only in T-ALL anti-CXCR4 monotherapy shows strong efficacy, suggesting a strong dependence of these tumor cells on CXCR4 signaling [6]. As relapse in T-ALL remains a challenging issue, these new data call for clinical trials to incorporate CXCR4 antagonists either as single agents following induction therapy, or as part of the first induction therapy regimen or later, during the consolidation phase.

In conclusion, the calcineurin/NFAT pathway acts as a fundamental bridge between microenvironmental-derived signals and T-ALL cells, mediating a complex crosstalk that is so far only partially dissected, but that already lead to the identification of novel targets of therapeutic relevance to T-ALL treatment.
